# Stretchability—The Metric for Stretchable Electrical Interconnects

**DOI:** 10.3390/mi9080382

**Published:** 2018-08-01

**Authors:** Bart Plovie, Frederick Bossuyt, Jan Vanfleteren

**Affiliations:** 1 Department of Electronics and Information Systems, Ghent University, Technologiepark 15, 9052 Zwijnaarde, Belgium; 2 IMEC vzw, Kapeldreef 75, 3001 Heverlee, Belgium; frederick.bossuyt@imec.be

**Keywords:** stretchability, electronic measurements, stretchable circuits, design metrics, reliability

## Abstract

Stretchable circuit technology, as the name implies, allows an electronic circuit to adapt to its surroundings by elongating when an external force is applied. Based on this, early authors proposed a straightforward metric: stretchability—the percentage length increase the circuit can survive while remaining functional. However, when comparing technologies, this metric is often unreliable as it is heavily design dependent. This paper aims to demonstrate this shortcoming and proposes a series of alternate methods to evaluate the performance of a stretchable interconnect. These methods consider circuit volume, material usage, and the reliability of the technology. This analysis is then expanded to the direct current (DC) resistance measurement performed on these stretchable interconnects. A simple dead reckoning approach is demonstrated to estimate the magnitude of these measurement errors on the final measurement.

## 1. Introduction

Stretchable circuit technology is a recent development, having seen the light of day over the span of the last two decades. It is an attractive solution to common design problems; that is, the need for an electronic circuit to cover large areas or to conform to a moving and deformable device, either during production or in use [1,2].

Before the development of stretchable circuit technology, this was usually solved using long spring-loaded cables, stored in an enclosure that fed and retracted the cable as was necessary to cover the distance [3]. This cable-based approach has the advantage of being incredibly reliable if implemented correctly; additionally, it works from direct current (DC) all the way up to the gigahertz range and offers the capability to handle large currents. However, for all these advantages, this technique has a significant drawback: it requires a large volume for even the smallest number of interconnects. An alternative method, which remains popular and still makes a frequent appearance, is the use of coiled flexible circuit boards to cover these transitions [4]. In fact, both methods are surprisingly common in consumer-applications (e.g., devices with retractable power cords and hard drive reading heads).

In comparison, modern-day stretchable electronics use a more elegant approach to provide high-density interconnects over short to medium distances [5]. These methods are often based on thin, flat substrates embedded in soft elastic polymers, and achieve stretchability through careful structuring or speciality materials [1,6]. These techniques are applied on all levels, ranging from pinhead sized semiconductor devices, all the way to circuit boards covering massive composite structures [1,2,4,7,8,9,10]. 

While some exceptions do exist, most technologies have one feature in common: the lumped circuit elements (e.g., resistors, LEDs, and transistors) remain rigid and non-elastic and are relegated to rigid “islands”; meanwhile, the connections between these islands are made stretchable [7,8,11]. This segregation is possible because most electronic circuits use highly modular designs, with the interconnections between these modules being less critical—meaning that modifying these connections does not affect the circuit’s functionality. As a result of this approach, the inherent capabilities of the circuit, regarding stretchability, are quantifiable by investigating the interconnections themselves, or at least that is the assumption commonly made. Arguments exist against this approach, but comparisons including the islands become difficult without considering specific circuit designs. Because of this reason, and this reason alone, only the interconnects themselves are considered in this manuscript, and for large high-density circuits, the effect of the islands should definitely be included.

The traditional approach to compare stretchable circuit technologies is based on considering the maximum percentage increase in length. This metric works by measuring the maximum increase in length the interconnect can survive, and dividing it by its original starting length—measured between the terminals [12]. This simple approach can be a fair indication of technological abilities when considering planar circuits using similar materials and designs, and is generally called “stretchability”.

Closely related is the percentage increase in resistance; this is the increase in resistance divided by the resistance measured between the terminals before stretching. It is relatively easy to couple these resistance increases to physical effects, for example, in metal meanders, an increase of resistance tends to indicate the presence of mechanical damage (e.g., necking and micro-cracks) [13]. Whereas in the case of conductive filler particles in a polymer matrix, it can indicate the average proximity of these conductive particles to each other [14]. Upon unloading the stretchable interconnect, which is to say the elongation is reduced to zero, the latter will recover, assuming no defects form in the polymer matrix and no conductive fillers migrate. So, a permanent increase in resistance here would mean the polymer material did not return entirely to its original length, or significant defects formed. The metal meander, on the other hand, will permanently maintain this damage, even though it might not be directly visible because of the metal being pressed together again, which creates an unreliable electrical connection [5,13].

Sadly, both these metrics can introduce a significant amount of bias towards a particular technology, just because of the way these values are measured. What may initially appear to be an insignificant measurement error can quickly add up and lead to incorrect results. To demonstrate the flaws of the above “stretchability” definition, we design, fabricate, and measure a circuit intended to exploit these flaws, and then define a series of alternative metrics to compensate for them. Additionally, the more subtle points of interconnect resistance measurements for stretchable electronics are highlighted.

## 2. Materials and Methods 

**Figure 1:**
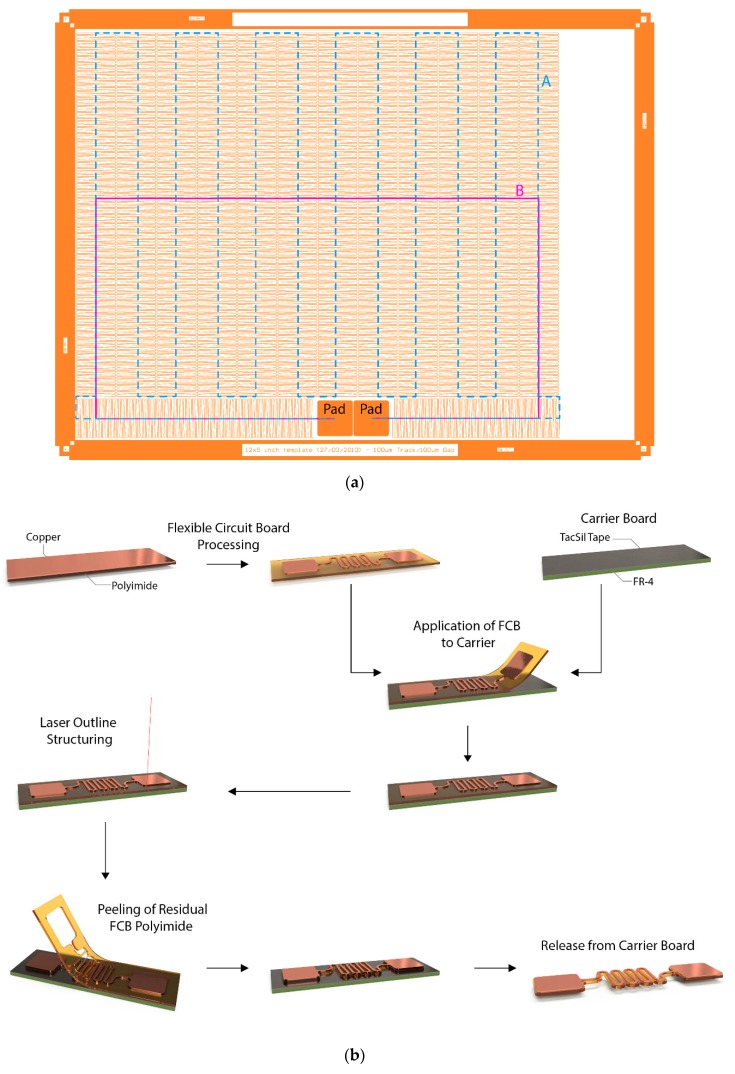
Manufacturing of free-standing stretchable circuits. (**a**) Copper artwork of the manufactured stretchable circuit. (**b**) Process flow used to manufacture the stretchable circuits. First, a flexible copper clad laminate is patterned using photolithography and wet etching. The resulting flexible circuit board (FCB) is then applied to an FR-4 carrier board covered with a pressure-sensitive TacSil Tape adhesive for mechanical support. The outline of the meanders and connection pads is then defined by cutting the flexible circuit board material using a laser cutter. The excess material surrounding the circuit is removed by hand, after which the circuit can be released from the carrier board by carefully peeling it off.

**Figure 2:**
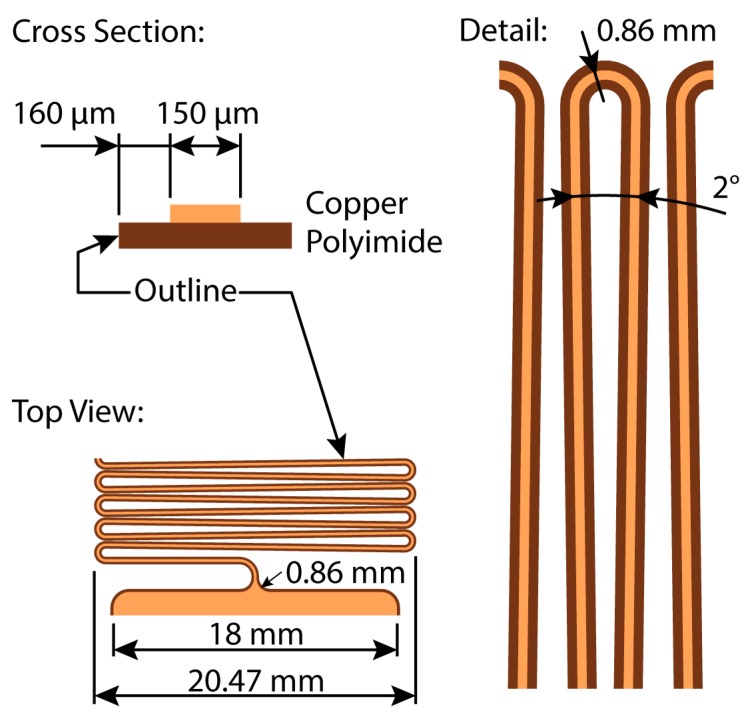
Design details of the stretchable circuit. The side of the polyimide track is the circuit outline. The meanders fold back on themselves at an angle of 2°, optimizing the amount of track in a given surface area without introducing extremely sharp corners.

The circuit was fabricated using a 246 mm by 207 mm piece of UBE Upisel-N SR-1220 (UBE EXSYMO Co., Ltd., Tokyo, Japan) polyimide flexible copper clad laminate (FCCL) with 18 µm of rolled-annealed copper on 50 µm of polyimide. Using standard printed circuit board processing techniques [4], photolithography and wet-etching, the FCCL was structured into a flexible circuit board (FCB), as illustrated in Figure 1b. This FCB was attached to a 1.6 mm FR-4 carrier board (Hitachi Chemical Company, Ltd., Tokyo, Japan) covered with Taconic FH20LB Tacsil tape (TACONIC, Seongnam-si, Republic of Korea)—a pressure sensitive adhesive for reflow assembly of flexible circuit boards—using a hand roller. The purpose of this adhesive is to form a tacky surface that will hold onto the FCB to prevent entanglement as it is structured into a long meandering (also sometimes referred to as serpentine [15]) interconnect. Once the FCB is attached, the carrier board is placed underneath a 10 kHz-5 W pulsed Nd/YAG laser cutter (OPTEC, Frameries, Belgium). The FCCL is cut at a rate of 5 mm/s without damaging the TacSil tape and carrier board underneath. The material surrounding the desired circuit was peeled away using tweezers and discarded, as shown in Figure 1b, leaving behind the circuit design shown in Figure 1a and Figure 2. 

The design of the stretchable interconnect, of which the design file is available as supplementary material, has the goal of optimizing the stretchability. The easiest way to improve the potential increase in length is having this length of conductor available, though this does not necessarily have to be in a practical location. Additionally, placing the pads next to each other increases the achieved stretchability without leading to any practical benefit. A third factor to consider is the reliability, because this is not included in the definition of stretchability, the design can use sharp corners with a small radius, which will hamper reliability. The first step is easily done by placing the 18 mm by 18 mm contact pads against each other. A critical value in this entire operation is the outline spacing, the distance between the edge of the copper artwork and the edge of the polyimide, as indicated in Figure 2. The minimum spacing for the outline of the flexible circuit board in regard to the copper is 100 µm; however, for yield reasons, the outline spacing was increased to 200 µm between the pads, resulting in a distance of 400 µm.

From these pads, a 150 µm wide copper trace leaves with an outline offset of 160 µm on both sides—as indicated on Figure 2. After the first 90° bend, a U-shaped meander was started, which is folded back on itself—meaning the straight sections connecting the arcs are no longer parallel. This meander has a pitch of 1.5 mm and a height of approximately 20.5 mm and was looped in an accordion pattern above the contact pads. Excluding the length of the contact pads, this meander spans a distance of 66,637.5 mm along its center line. Two additional center lines were defined in Figure 1a, the purpose of which will become clear later on. The first centerline (A), which closely follows the zig-zag pattern, has a length of 2711.7 mm; the second one, however, does not follow this pattern and has a length of only 639.1 mm. The total surface area used by this circuit, including the contact pads, is 50,905.0 mm^2^—a number that will become important later on.

**Figure 3:**
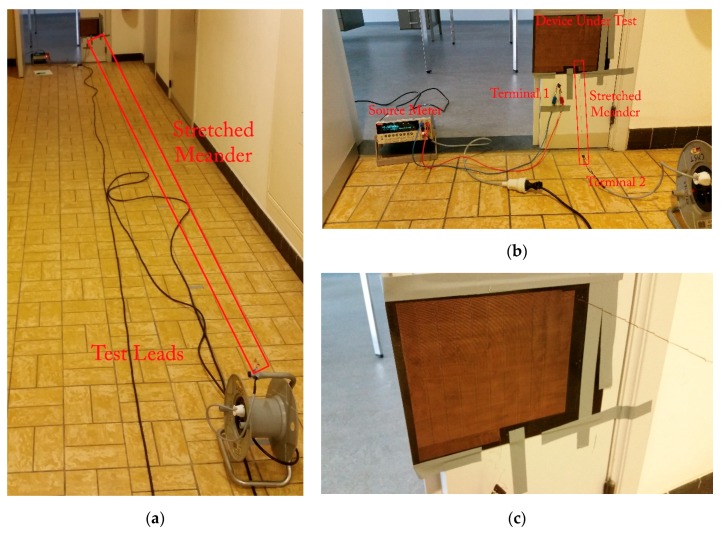
Measurement setup used to determine the meander resistance as a function of the extension: (**a**) measurement setup at the 10 m extension mark; (**b**) measurement setup at the start of the measurement before any extension. The cable reel visible at the bottom right of the image was used to extend the test leads to the desired length; (**c**) detailed view of the carrier board and circuit at the 10 m extension mark.

Conventional tools, such as a tensile tester, are unable to deal with meanders of these lengths, meaning standard test methods were impossible to use. Instead, the carrier board with the circuit was attached to rigid surface perpendicular to the floor in a long corridor and markings were put on the floor at one-meter intervals using a tape measure, as shown in Figure 3b,c. To determine the resistance of the meander, a Keithley 2400 source meter (Tektronix, Inc., Beaverton, OR, USA) was set up to perform a four-wire measurement with a test current of 1 mA. To extend the test leads, a conventional power cable reel, visible in Figure 3a, was used. To ensure mechanical stability during extension of the meander, one of its pads was attached to the cable reel, as seen in Figure 3a. The reel was then moved backwards at a rate of approximately 10 cm/s to extend the circuit to the desired lengths. Before measurement, the resistance was allowed to stabilize for at least 30 s to eliminate possible measurement errors due to mechanical vibrations.

**Figure 4:**
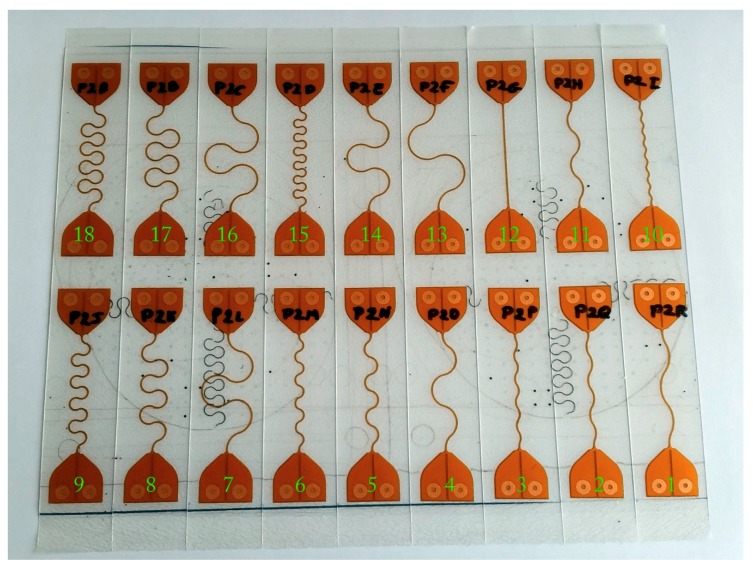
Meander test samples encapsulated in thermoplastic polyurethane using a vacuum press. The black debris visible on the sample originates from FCBs previously cut on the same carrier board.

A second experiment, demonstrating the design dependence of stretchability—even within a given technology—uses a variety of stretchable interconnect designs. For this purpose, 18 designs based on flexible circuit board technology were manufactured and tested until failure using a tensile tester (Instron 5543, Instron, Norwood, MA, USA). As before, the flexible copper clad laminate (Shengyi SF305 101820SR—25 µm of polyimide with 18 µm of adhesively bonded copper) was patterned using industry standard practices. Afterwards, a coverlay film (Shengyi SF305C 1025) was bonded to the resulting flexible circuit board using the manufacturer’s recommended press program in a vacuum press (Lauffer RLKV25). The FCB was placed on an identical carrier board and laser structured using a picosecond pulse-length Nd/YAG laser system (3D Micromac microSTRUCT™ vario) to define the meander outline—as illustrated in Figure 1b. The spacing between the copper track, which is 100 µm wide, and the laser path was set to a rather hefty 300 µm—representing the minimum tolerances achievable in an industrial setting. Some of the meanders were encapsulated between two layers of thermoplastic polyurethane (TPU)—100 µm Covestro Platilon U4201 AU—using a vacuum press. To ensure even pressure distribution during the lamination of the TPU, a 1/8-inch silicone–rubber–foam press pad (Rogers Corporation BISCO Foam HT-870) was used between the 25-µm polytetrafluoroethylene (PTFE) release foil and separator plate. The press applies 10 N/cm^2^ at 175 °C for 20 min in a <2 mbar vacuum atmosphere to bond the TPU layers to the polyimide and together. The final samples, shown in Figure 4, were then cut using a guillotine cutter. To enable electrical measurements, the TPU was opened up using a soldering iron, and flexible wires were attached to the samples using leaded solder (Sn_63_Pb_37_ alloy).

**Figure 5:**
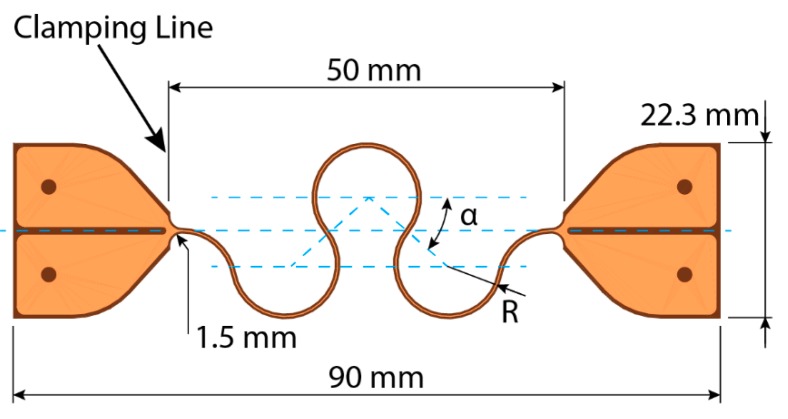
Design of the test vehicle with horseshoe-shaped meanders used for the tensile tests. R is the radius of the horseshoe-shaped meander segment, while α is the opening angle. These two variables combined completely define a horseshoe-shaped meander. To provide a smooth transition to the contact pads, a small 1.5-mm fillet was introduced at both ends. The contact pads at either side of the meander were split up to enable a four-point measurement with separate drive and sense lines.

Each meander has an effective length of 50 mm between the contact pads, of which 3 mm is taken up by the fillets to transition from the connection pads to the meander, as shown in Figure 5. These connections pads at either end of the meander are necessary for both clamping and electrical connectivity—to perform a four-point measurement while the meander is being stretched in a tensile tester. The contact pads were split up and only meet near the start of the meander in an attempt to eliminate their influence on the measurement. The design parameters of these meanders are listed in Table 1, all except design #12 are of the horseshoe-shaped meander type. Design #12 is a reference with a straight track in between the connection pads to determine the inherent capability of the material to meet these deformations. A schematic representation of such a test sample with relevant measurements is visible in Figure 5. Alternatively, the original design file is available as Figure S2. 

**Figure 6:**
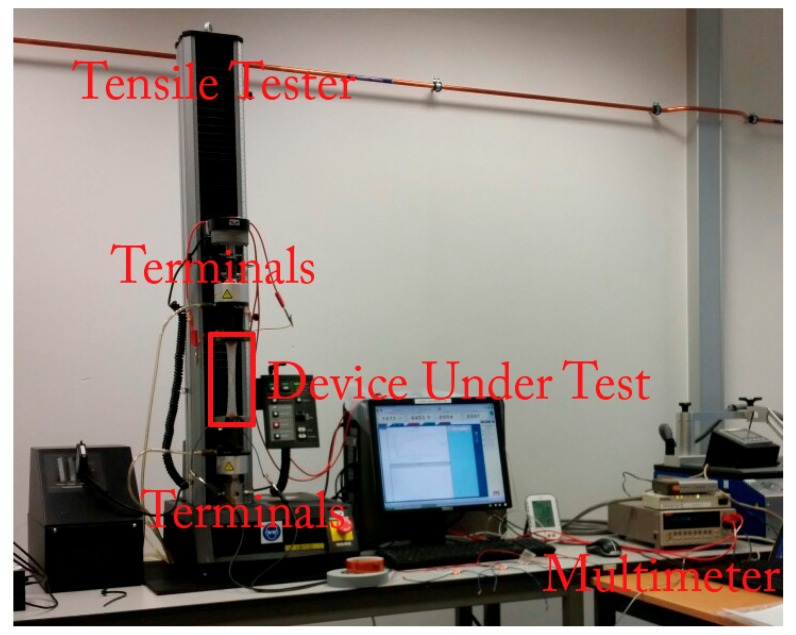
Tensile tester (Instron 5543) with the benchtop multimeter (Keithley 2001) performing the electrical measurements at the right-hand side. The multimeter was triggered over a network connection, and data were recorded using a secondary computer.

The encapsulated samples were clamped in the tensile tester—shown in Figure 6—and elongated at a rate of 0.5 mm/s until failure. At the same time, the DC resistance of the samples was measured using a benchtop multimeter (Keithley 2001), which was triggered by the tensile tester software using a network connection. For this, it was set to auto-ranging with an upper limit of 200 Ω and averaging over 10 measurements—with one measurement per cycle of the 50 Hz power grid—because only the estimated point of failure was of interest, not the precise resistance increase, to determine the point of failure. 

## 3. Results

**Figure 7:**
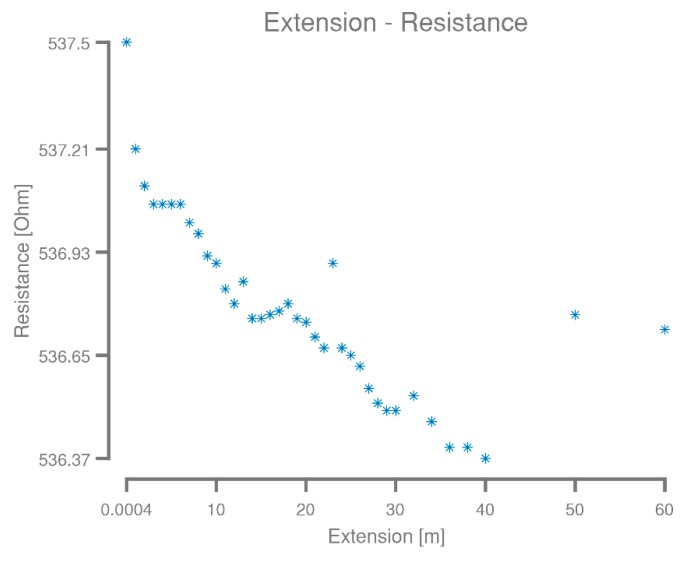
The resistance of the 66.6 m long meander plotted as a function of its extension. A noticeable, but procentual small drop in resistance was observed as the meander was extended, most likely due to the copper folding back on itself at each arc, which would slightly reduce the electric path length when stretching the meander.

The meander started out with a resistance of approximately 537 Ω, while it was flat on the carrier board, verified using a zeroed Keysight U1253B multimeter (Keysight Technologies, Santa Rosa, CA, USA). After mounting in the measurement setup, as shown in Figure 3b, this resistance increases slightly to 537.50 Ω, as measured with the Keithley 2400 using a four-wire measurement. The observed resistance as the meander was extended is shown in Figure 7.

A slight drop in resistance from 537.5 Ω to 536.37 Ω was observed during the experiment, contrary to what might be expected from a meander under significant strain. However, this can most likely be explained by the arcs at the end of the U-shaped meanders twisting and folding back on themselves as the meander extends, something slightly visible in Figure 3c. Extension of the meander was stopped at sixty meters because of length limitations of the corridor, limiting the achieved stretchability to 15,000,000%. When attempting to roll the meander on a spool for storage after the experiment, it broke in several places at the points where it twisted during the elongation. More importantly, this demonstrates the design dependence of the term stretchability. 

The chosen distance between the start and end point is poorly defined and allows for parlor tricks as demonstrated above. Possible values for this circuit are the width of the cut between the pads (70 µm), the distance between the nearest copper features (400 µm), the distance between the centre of the pads 18.4 mm, the distance between the meander starting points (36.4 mm), or any other ill-devised definition. With each distance leading to its respective extension: 85,714,200%, 14,999,900%, 326,100%, and 164,800%. 

The two center lines, A and B, defined in Figure 1a, provide a more realistic length, leading to 2113% and 9288%, respectively. While in this case, center line A provides a more accurate number, the choice between these two center lines can become quite tricky in some instances. For example, using center line B might be justifiable if the radius of 0.86 mm (Figure 2) is increased to 10 mm. In short, defining the initial length against which stretchability is measured has a considerable influence on the result. However, making the *“correct”* choice might not be trivial in some cases.

Needless to say, unless the same definition and measurement guidelines were used, any comparison between these numbers is meaningless. Additionally, this meander will fail whenever the tension on it is released, making it useless for most applications. Clearly, a better definition of stretchability is required to remove ambiguity. 

### 3.1. Alternative Stretchability Metrics

Defeating stretchability as a metric of a stretchable interconnect’s capabilities is trivial. As demonstrated above, it only requires creating a long wire using the technology at hand. A useful metric is unbiased and considers the reliability of the technology at hand. Solving this for the case of stretchable electronics seems trivial at first.

For a planar substrate, dividing the maximum achieved elongation by the occupied surface area before elongation would prevent looping the wire around the contact pads, as was done here. In fact, this planar stretchability (PS) should provide a fair assessment of a technology’s capabilities at first glance. The maximum PS would depend solely on the minimum feature size and not the design; some small gains could be made by shrinking the contact pads; however, these would be minimal.

Planar Stretchability = Maximum Elongation/Area(1)

This, of course, does not account for technologies that use out-of-plane features to create a stretchable interconnect [10,16]. These could create a tower of wire that might take up a relatively large volume while providing no cyclic endurance, like the above circuit—a single elongation might break the interconnect. An easy method to alleviate this is to include the thickness of the circuit by dividing the maximum elongation by the volume of the bounding box around the stretchable interconnect, leading to the volumetric stretchability (VS):

Volumetric Stretchability = Maximum Elongation/Volume(2)

To eliminate the effect of the units, each value is normalized to millimeters; alternatively, the unit used could be mentioned next to the variable as a subscript (e.g., PS_mm_). At first glance, this might result in a constant number for a given technology. However, the design of the interconnect still depends on electrical parameters, such as impedance and maximum current carrying capability, and the mechanics of the encapsulating material.

Using these metrics and looking back at the above example, consider the 60 m extension, 50,905.0 mm^2^ surface area, and 3461.5 mm^3^ volume (68 µm thickness). The planar stretchability then becomes 1.178 and the volumetric stretchability becomes 17.334. These values are significantly more useful; the required substrate surface area or volume is directly related to the cost to fabricate the device. Additionally, merely dividing the desired final length by the achieved PS or VS will indicate the required area or volume to achieve a given wire length. However, this still would not help determine the actual stretchability a designer or engineer might expect from a circuit—the above circuit is still frightfully unreliable, even though it might score well in a comparison.

Defining reliability as a single number is a troublesome prospect. However, for meanders, a few assumptions are possible. For example, it is possible to postulate the following: “Each directional change between segments of a conductor in a stretchable electrical interconnect where the angle between individual segments exceeds 30 degrees is prone to failure over time”. This statement is most certainly wrong in an absolute sense, but provides a straight-forward method to quantify the chance of failure by cutting the stretchable interconnect into segments. 

Providing a generic definition for a segment is troublesome; however, using the above definition, a few cases can be defined. First, any sharp corner (<90°) between two straight lines would most definitely be a transition at risk of failure. A second case is an interconnect that consists out of multiple identical elements that are repeated to form an interconnect. The third case, which is close to the above definition, would be a generic catch-all case; consider the tangent along the interconnect its center line. If this tangent changes more than 30° versus the tangent at an earlier point on the line this, indicates a transition, and hence a segment. Under ideal circumstances, the first two definitions are used, but these are troublesome to apply to special cases (e.g., fractal meanders).

Consider a segment with a known length, and n segments are required to span this distance. Neglecting the start and end points, the chance of failure for each segment and each transition between individual segments ris P_1_[i] and P_2_[i], respectively, where i is the cycle number during the cyclic test. Assuming the segments are independent of each other, with an equal chance of failure, and the applied strain is irrelevant, the chance a meander segment will survive a certain stretch cycle becomes the following:

P_Survival_[i] = (1 − P_1_[i])(1 − P_2_[i]) (3)

Hence the chance of failure becomes the following:

P_Failure_[i] = 1 − (1 − P_1_[i])(1 − P_2_[i]) (4)

Taking a few statistical liberties, like assuming the stretch cycles are completely independent of each other, the chance a meander segment breaks after i cycles becomes the following:

P_F_[i] = 1 − Π ^i^_j=0_ (1 − P_1_[j])(1 − P_2_[j]) (5)

While this might appear extreme at first glance, the survival chances P_1_ and P_2_ are close to 1 in most technologies, resulting in a rather small P_F_. Assuming each meander segment is independent of its neighbors, the chance of failure in cycle i for n segments then becomes the following:

P_F_[i, n] = 1 − (Π ^i^_j=0_ (1 − P_1_[j])(1 − P_2_[j]))^n^ (6)

Clearly, an increase in n will increase the chance of failure. However, this rudimentary approach fails to take into account the strain the meander might experience; but it does demonstrate the effect of the number of segments or transitions n on meander reliability if these are considered weak points. Introducing this into the stretchability metric is trivial; simply dividing the stretchability by n ought to penalize a technology with many transitions. This leads to the definition of compensated planar stretchability (CPS) and compensated volumetric stretchability (CVS):

Compensated Planar Stretchability = Maximum Elongation/(n } Area)(7)

Compensated Volumetric Stretchability = Maximum Elongation/(n } Volume)(8)

In both cases, the area and volume are those for the complete meander. The above circuit has a staggering 4033 transitions, which meets the above requirement, slicing the CPS and CVS down to 0.003 and 0.004. For the case of a polymer matrix filled with conductive particles, setting n equal to one is an acceptable choice, unless it is patterned as well, because the likeliness of failure will depend on the material’s inherent properties instead of the interconnect design.

An even more generic approach would be to consider the chance of failure occurring per length unit after i cycles P_FPL_[i]. If such a number were available, it could easily be included by substituting n by (1 − P_FPL_[i]). However, this would only provide a momentary comparison point during cycle i. A more generic approach would distill the reliability function P_FPL_[i] into a single number based on the number of life-cycles a device should survive. 

Let i_expected_ and k be the number of stretch-cycles the device should survive during regular use and the percentage of acceptable failures within this period, respectively. While setting k to zero might be an attractive prospect, no economical process achieves a 100% yield. Consider the function f[i] that returns the percentage of failed devices after i cycles; multiplying it by a weighing function and calculating the sum over the entire range of i would return a single number that signifies the reliability. This weighing function should heavily punish crib deaths (early failures), while not significantly penalizing for failures beyond i_expected_. Based on this, we can propose the weighing function w[i]:

w[i] = a } exp(−b } i) ∧ ∀i ∈ ℕ: w[i] > 0 ∧ a, b ∈ ℝ^+^_0_ ∧ ∑^+∞^_i= iexpected_ w[i] = 1. (9)

The latter condition in Equation (9) ensures failures after the expect lifetime do not significantly count towards the (un)reliability metric. However, a more reliable technology should still achieve a better result. 

Next, factoring in the acceptable percentage of failures is done by the following:

a = 1 − k(10)

Once a is known, determining b is trivial by solving the following equation numerically:

exp(b) − 1 = (1 − k) } exp(b − b } i_expected_) (11)

The reliability metric R[i_expected_, k] can then be calculated by multiplying the weighing function point-wise with the cumulative failure percentage given by f[i]:

R[i_expected_, k] = ∑^+∞^_i = 1_ (1 − k) } exp(−b } i) } f[i] (12)

Using this metric, a higher number will indicate a less reliable technology, meaning it can substitute the compensation factor in Equations (7) and (8).

The exact method used to compare stretchable interconnect technologies and designs should be selected based on the application, available data, and desired outcome. For example, a smart health monitoring patch would only be expected to last a day, while a consumer device in the European Union would have to last for over two years. Sadly, calculating these metrics is infeasible at this time because of insufficient data and will most likely only happen at the point of large-scale industrialization. 

### 3.2. Example Case

Analysing the data from the second test using the above methodology—planar stretchability—demonstrates the usefulness of these modified metrics and their potential pitfalls. Per design, four samples were tested, two with and two without TPU encapsulation. Table 2 and Table 3 list the mechanical and electrical measurements, respectively. As mechanical failure, the first point at which the material ruptures was chosen, while electrically, a ten-fold resistance increase versus the starting resistance was used. Because of time uncertainty between the trigger signal being sent and the start of the measurement, one millimeter is deducted from the extension before electrical failure.

**Figure 8:**
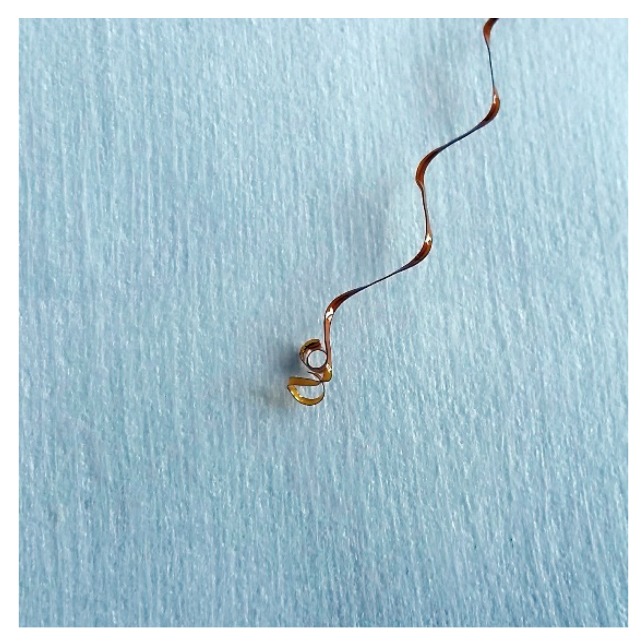
Delamination of the coverlay and curling of the FCB materials after tensile tests performed on the interconnects.

The observed failure (Run 2—Design #13) in the mechanical measurement occurred because of a loss of air pressure to the pneumatic grips during the test, releasing the sample. The more common failures during the electrical measurement were caused by a variety of problems between synchronizing the mechanical and electrical measurements. In both cases, the actual achievable elongation values are expected to be less, the reason for this is a combination between the sample slipping and cantilevering in the claws. Measuring the exact length after failure was impossible because of delamination and curling of the material, as shown in Figure 8.

Averaging the values for both the free-standing and encapsulated cases, and separating them into mechanical and electrical failure, the stretchability and planar stretchability were calculated for each value, resulting in Table 4. The considered area when calculating the planar stretchability is the actual width the interconnect takes up on the sample multiplied by 47 mm; the values used are listed in Table 1. The starting fillets were neglected, as these are identical in all cases. However, when comparing technologies, or if they differ between designs, these fillets or other transition structures should be included.

The straight track reference (Design #12) appears to have a stretchability of 20% to 30%. This high stretchability is not practical because it originates from plastic deformation of the material, combined with the above clamping problems. Additionally, it is non-reversible, limiting its use to one-time deformations. As a result, this should only be considered a baseline measurement.

**Figure 9:**
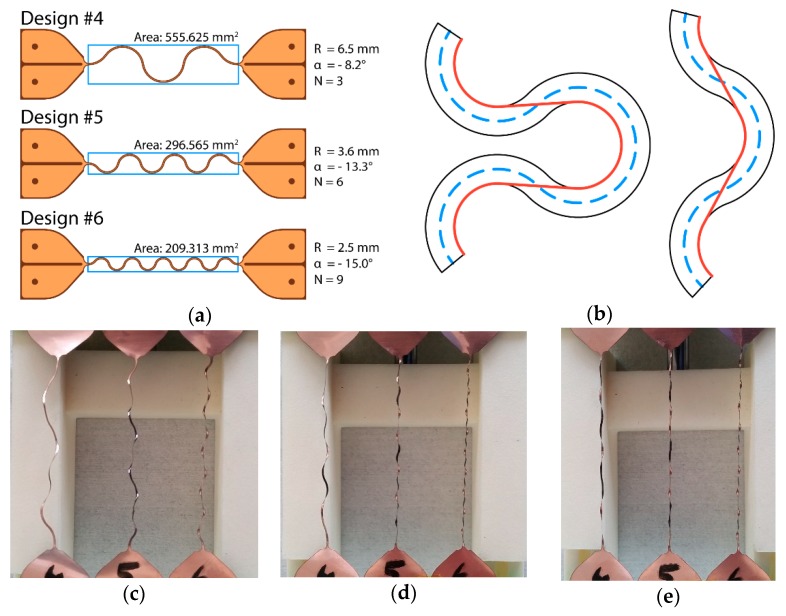
Detailed view of some of the tested samples. (**a**) Parameters and comparison of Designs #4, #5, and #6, illustrating the clear distinctions between designs with similar path lengths. (**b**) The length along the centerline (dashed line) is not the actual length a meander can traverse without undergoing plastic deformation. Instead, the shortest possible distance that traverses the meander track provides a more accurate value. (**c**) Three dummy samples of Design #4, #5, and #6 with a 5 mm extension applied. (**d**) Three dummy samples of Design #4, #5, and #6 with a 10 mm extension applied. (**e**) Three dummy samples of Design #4, #5, and #6 with a 15 mm extension applied.

Considering the possible deviation caused by plastic deformation and the test (20% to 30%), the experimental stretchability values are closely in line with the theoretical values calculated by considering the meander’s centerline—as expected. However, the planar stretchability (PS) values—multiplied times a thousand for the sake of convenience—tell a different story. The clearest example is design #4; while it achieves the same stretchability as designs #5 and #6, it requires significantly more surface area to do so, as illustrated in Figure 9a, making it less attractive from a manufacturing cost perspective.

At the same time, the fallacy of the planar stretchability is magnified by the reference design, which scores a staggering 284 to 438—three to ten times higher than the meanders. This is not surprising, because a straight line is the most efficient way to connect two points on a flat substrate. However, a wider trace would lead to a lower planar stretchability; luckily, the higher stretchability meanders would experience a similar drawback, because they would have to use even wider copper to achieve the same resistance as the much shorter straight trace. However, this method heavily promotes horseshoe-shaped meanders with small radii and a large number of segments because it packs a lot of conductor length per substrate area, even though smaller bending radii might not necessarily be an advantage from a reliability perspective.

The compensated planar stretchability (CPS), listed in Table 5, changes this figure entirely by introducing the number of transitions or segments—available in Table 1. This is, once again, exceptionally clear when comparing designs with similar stretchability, such as Design #4, #5, and #6. While #5 and #6 were previously closely matched because they take up similar substrate surface areas, #4 and #5 now have the advantage because they have less segments. This stands to reason because the increased space between the individual segments would allow more encapsulation material in-between the segments—meaning it can deform more before developing tears in the encapsulation material. An additional reason that Designs #4 and #5 are preferable over #6 can be seen in Figure 9c; at 5 mm elongation, Design #6 is almost entirely stretched, while #4 and #5 still have some headroom. This situation only worsens as the elongation is increased to 10 mm (Figure 9d) and then to 15 mm (Figure 9e). While this might appear counterintuitive at first glance, the reason for this discrepancy can be found in Figure 9b. The length along the meander centerline, as listed in Table 1, is not the actual length a meander can achieve without plastic deformation. Instead, the smallest radii in combination with a tangent running in between these radii provides an accurate number. As a result, the meander with a small number of segments, and hence a lower opening angle α, can potentially span a far longer distance without undergoing plastic deformation.

Instead, the smallest radii in combination with a tangent running in between these radii provides an accurate number. As a result, the meander with a small number of segments, and hence a lower opening angle α, can potentially span a far longer distance without undergoing plastic deformation. For example, when considering the difference between the centerline and this actual length on a per segment basis, Design #4 only loses 4.56% of its length per segment, while this increases to 7.49% and 10.10% for Designs #5 and #6, respectively, explaining the results seen in Figure 9.

The advantages of compensated planar stretchability are also apparent when comparing Designs #16, #17, and #18. While Designs #18 and #17 should significantly exceed the stretchability of Design #16, both their CPS figures are significantly scaled down by introducing the number of segments as a factor. Given that the minimum spacing between two separate meander segments decreases from 5.038 mm to 2.831 mm, and finally to 1.849 mm, respectively, for these three designs, this is a correct figure; especially when considering a larger radius should decrease the strain experienced by the copper.

### 3.3. Resistance Measurements

DC resistance measurements are the golden standard to electrically verify stretchable electronics. The general idea consists of proving the prowess of the technology or design at its intended application; that is, passing an electrical signal. However, many measurements downplay the actual effects observed or are fundamentally flawed from the start by failing to consider realistic use conditions.

The above circuit was tested using a 1 mA current, a typical test current for resistance measurement to place the returned voltage in the multimeter’s 2.1 V measurement range. However, if the function of the device was to carry a large current (e.g., 10 A), the performed measurement would be pointless because the heating might significantly affect the mechanical reliability of the circuit. Carrying large currents will pose different challenges as opposed to, for example, capacitive sensing, the measurement should use a test current close to the one encountered in the intended application whenever possible. This is especially important when considering the failure mode of conductors can change dramatically as the width and thickness increases, as commonly seen with flexible circuit boards, meaning a small dimensional change to improve electrical performance might cause significant issues mechanically.

**Figure 10:**
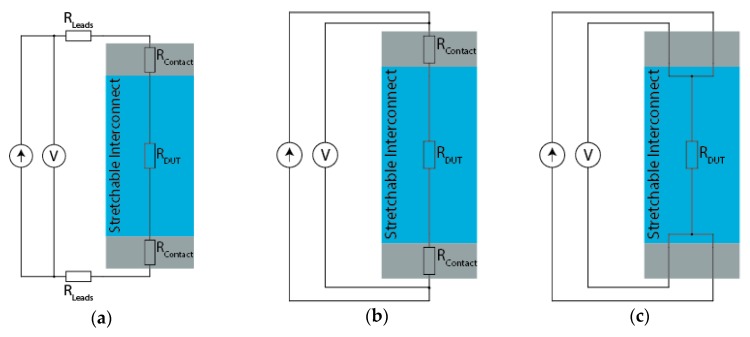
Measurement setups for resistance measurement: (**a**) two-wire setup affected by lead and contact resistance; (**b**) quasi four-wire setup affected by the contact resistance; and (**c**) true four-wire setup that eliminates the effect of lead and contact resistance.

Under any condition, the first step is correctly measuring the observed resistance. Three circuit topologies, shown in Figure 10, are in everyday use when measuring the resistance of an electrical interconnect. The first topology (Figure 10a) is equivalent to placing multimeter probes on the contact pads and performing a two-point measurement. Here, both the contact and lead resistance come into full effect. The next possible topology is the quasi four-wire approach (Figure 10b), where separate wires are used for driving the measurement current and sensing the voltage, but they are connected before contacting the device-under-test (DUT). As a result, the contact resistance is still present. The final case, a true four-wire measurement (Figure 10c), is the only correct method in most cases [17,18].

The method in Figure 10a is only suitable for quick process verification, secondary checks, or when dealing with resistances of multiple kilo-ohms. For the tested sixty-meter meander, Figure 10b presents the minimum acceptable measurement. The movement of the test leads, and especially the coiling on the reel, might significantly affect the resistance of the test leads during the measurement. However, during movement, the contact resistance can change dramatically, making the true four-wire measurement the only correct method.

To understand the importance, it is worth looking at ballpark figures. Consider a stretchable interconnect in an undefined technology; this hypothetical copper-based interconnect has a resistance of 300 mΩ. A two-wire measurement (Figure 10a) is performed on the interconnect while it is stretched, hoping to monitor the formation of micro-cracks over time. The test leads have a resistance of 100 mΩ each, while the nickel-plated probes have a contact resistance of 25 mΩ with interconnect. Before stretching the interconnect, the multimeter will see 550 mΩ, which, for example, increases to 700 mΩ when the interconnect is fully extended—or a 27.3% increase. While actually, the resistance increase the meander experienced was 50%, a significantly different result. 

The next measurement, performed by a slightly more skilled operator, uses a zeroed multimeter—meaning the probes were placed on the same contact pad to make a reference measurement. This action will indeed subtract 250 mΩ from the measured resistance and provide a 50% figure in theory. However, in practice, the measurement is still flawed and the formation of small micro-cracks will still be lost to the measurement noise and error. The movement during stretching will slightly change the resistance of the test leads, and shifting of the contacts will result in a slight, but noticeable alteration of the contact resistance because it is a function of pressure, surface condition, and location [19,20,21]. The magnitude of this effect is difficult to estimate as it heavily depends on the surface roughness and type of probe. However, it is safe to say it will create an uncertainty in the milliohm range or larger (e.g., ±5 mΩ). Additionally, the multimeter might not be in its optimal measurement range; the software will dutifully subtract the 250 mΩ and present a 150 mΩ in ideal circumstances. However, if the measurement ranges were, for example, 500 mΩ and 5 Ω with a resolution of 1 mΩ and 10 mΩ, respectively, the measurement uncertainty would increase tenfold for this scenario—because the multimeter is now operating in its 500 mΩ range. This means that it is crucial to consider the measurement range the multimeter is in, and not blindly base the result on the displayed value, especially when dealing with low-cost handheld units.

Of course, not every effect comes into play in every situation; therefore, it is essential to understand the magnitude of the problem for a specific situation. Micro-cracks are initially virtually impossible to detect electrically; this is easy to prove using simple electrical theory. The resistance of a rectangular conductor at DC depends on its resistivity ρ, length L, thickness t, and width w [22]:

R = ρ } L/(t } w).(13)

Consider a copper trace with a thickness of 18 µm and width of 100 µm at 20 °C, it will have a resistivity of 16.8 nΩm [23,24]. If the slice in which the micro-crack occurs has a length of 1 µm, it will start with a resistance of 9.3 µΩ. To add 1 µΩ of resistance, the crack has to propagate 9.7 µm into the trace, and another 7.95 µm to add another 1 µΩ. In short, detecting micro-cracks early requires exact high-resolution resistance measurements. This also explains why failures might appear suddenly; the trace might have broken most of the way before the actual resistance increase becomes noticeable. Of course, detecting a single micro-crack tends to be impossible under most circumstances, luckily, they tend to occur in large numbers. However, based on this, we can conclude analyzing the mechanical condition of a circuit based on electrical measurements is a precarious method unless the measurement limitations are considered.

What is also interesting to study is the effect of this localized resistance increase when carrying larger currents. Consider, for example, the case of a constant current LED driver supplying 100 mA through the above 18 µm by 100 µm long interconnect. Assuming the interconnect spans a distance of 200 mm, it will have a resistance of 1.867 Ω and will dissipate approximately 18.7 mW over its entire length—a trivial amount not worth mentioning for a highly conductive material like copper. However, at the location of micro-cracks, things can turn for the worse quickly; a 1-µm long slice will dissipate only 93.33 nW, but this goes up to 933.3 nW for a 90-µm wide crack. Considering this slice weighs only 16.13 ng and copper has a specific heat capacity of 384.4 J/(kg K) [24], it requires only 620 nJ to increase its temperature by 100 °C—or less than one second at this current assuming no thermal energy is conducted to the surrounding material. While conduction to the surroundings will dampen the temperature rise, it does point out that thermal degradation of the encapsulating material, which supports the interconnect, can occur at the site of failing conductors. It is not far-fetched that this might significantly aggravate the situation and lead to earlier failures depending on the encapsulation material.

Another effect worth considering when measuring small resistances is the Seebeck effect, meaning any metal-to-metal junction starts behaving as a thermocouple [17]. In most measurement setups, this effect is trivial and readily eliminated by using a parallel measurement path on both sides of the sample, because the entire setup is at the same temperature. Alternatively, specific alloys can be used on the contacts to limit this effect further—usually labelled as “low thermal electromotive force (EMF)” by the manufacturer. However, heating caused by the circuit or mechanical movement can upset this isothermal environment and result in the introduction of large thermal voltages.

Consider a typical nickel-plated probe; the copper–nickel Seebeck coefficient is 10 µV/K for pure copper and nickel [25]. A temperature gradient of 10 °C will then lead to a thermal voltage of 100 µV. Using the initial example once more, a source meter sends a 100 mA measurement current through 300 mΩ, which results in a voltage of 30 mV over the sense leads in ideal conditions. The 100 µV offset by the thermal voltage skews this measurement to 301 mΩ, a relatively small error. The problem stems from the fact that many handheld multimeters are incapable of providing high test currents, unlike source meters or high-end bench meters, which will happily provide higher test currents. For example, the Keysight U1253B uses a current of 1.04 mA, which would result in a 312 µV output, adding 100 µV onto that results in a 400 mΩ value on the display (10 mΩ resolution). An even worse situation exists when considering copper–copper oxide contacts, which can exhibit Seebeck coefficients of 1 mV/K [17]. For this reason, choosing the correct probe for the intended application is of vital importance when measuring small resistances.

A final consideration is the time it takes the multimeter to perform the measurement. This measurement is rarely instant because the sample-and-hold circuit requires time to charge a capacitor [26]. A changing resistance during this interval—called the aperture time—is a potential cause for error, meaning movement of either the probes or the sample can introduce an unintended bias to the measurement [18]. This is exceptionally clear when attempting to eliminate the noise of the power grid by using aperture times that are multiples of 20 ms or 16.7 ms to average one or more power cycles. Ideally, the mechanical measurement stops, triggers the multimeter, and waits for it to complete a measurement before continuing the test.

In short, measuring small resistance changes requires a correctly configured measurement setup. The most substantial errors can be avoided by using a four-wire measurement with appropriate test currents to emulate self-heating. Samples should have clean electrical contacts at the same temperature, and the measurement instrument has to be configured to the correct range with a suitable test current.

## 4. Discussion

Stretchability is commonly used to describe the performance of a stretchable electronic interconnect. However, as demonstrated above, it is an unreliable metric for the performance of a technology because of its design dependence. A series of alternative metrics taking into account the area, volume, and reliability of the interconnect were proposed to limit the influence of the design. Additionally, the considerations for accurate resistance measurement were highlighted using simple examples, providing a straightforward way to estimate their importance for typical stretchable interconnect measurements.

## Figures and Tables

**Table 1:** Design parameters for the tested interconnects.

Design	Length [mm]	Radius [mm]	Opening Angle α [°]	Area [mm^2^]	Segments	Theoretical Stretchability [%]
1	50.605	10.000	−47.330	281.747	3	8
2	49.516	6.547	−56.166	137.118	6	5
3	49.506	4.555	−56.892	102.417	9	5
4	62.037	6.491	−8.238	555.625	3	32
5	62.046	3.649	−13.373	296.565	6	32
6	62.044	2.532	−14.995	209.313	9	32
7	97.147	6.678	39.037	1056.007	3	107
8	97.063	3.729	29.092	553.856	6	107
9	97.192	2.584	26.249	383.209	9	107
10	49.517	2.652	−57.394	72.190	16	5
11	52.029	6.999	−41.517	254.722	4	11
12	47.000	N/A	N/A	32.900	1	0
13	78.669	8.372	−90.000	1201.706	2	67
14	86.447	6.382	28.966	923.338	3	84
15	104.171	1.891	29.001	296.834	13	122
16	104.732	6.927	45.000	1144.398	3	123
17	124.688	4.262	45.000	716.767	6	165
18	133.551	3.078	45.000	526.740	9	184

**Table 2:** Mechanical measurements performed on meanders using a tensile tester.

	Free Standing				Encapsulated			
	Run 1		Run 2		Run 3		Run 4	
Design	Force [N]	Ext. [mm]	Force [N]	Ext. [mm]	Force [N]	Ext. [mm]	Force [N]	Ext. [mm]
1	7.46	17.3	7.42	17.5	31.65	17.1	37.05	19.1
2	7.66	18.7	7.85	17.9	33.93	18.1	34.02	15.6
3	7.49	16.8	7.51	16.7	34.41	18.0	34.96	16.7
4	6.69	24.7	7.01	29.8	34.71	30.2	35.81	21.1
5	7.41	31.7	7.54	32.2	36.40	25.7	37.20	23.6
6	7.42	31.7	7.29	29.5	33.58	21.6	37.38	24.5
7	8.06	80.2	8.06	79.9	35.85	28.7	35.99	34.3
8	7.03	67.3	7.62	73.8	35.05	34.7	35.52	41.4
9	6.84	64.8	4.67	55.8	36.85	41.2	39.50	45.7
10	6.72	12.7	6.80	13.0	32.00	14.8	28.55	14.4
11	7.82	22.4	7.98	25.1	34.99	21.4	34.47	20.3
**12**	**7.56**	**14.7**	**7.58**	**14.1**	**34.30**	**16.2**	**31.22**	**14.1**
13	7.60	52.7	Failure	Failure	36.01	27.2	33.58	26.6
14	7.91	70.0	7.69	52.9	38.88	35.7	35.56	29.2
15	5.39	64.4	5.20	64.0	39.08	48.8	37.83	44.5
16	7.94	89.1	7.97	84.8	35.70	31.8	35.62	28.2
17	7.57	107.7	7.57	101.9	39.61	47.3	36.71	38.4
18	6.98	106.0	7.03	108.4	30.01	48.4	37.24	49.5

**Table 3:** Electrical measurements performed on the meanders during the tensile test.

	Free Standing				Encapsulated			
	Run 1		Run 2		Run 3		Run 4	
Design	R_Start_ [Ω]	Ext. [mm]	R_Start_ [Ω]	Ext. [mm]	R_Start_ [Ω]	Ext. [mm]	R_Start_ [Ω]	Ext. [mm]
1	0.527	14.5	0.515	16.5	0.521	14.1	0.502	12.2
2	0.509	15.4	0.498	14.3	0.508	11.5	0.496	9.1
3	Failure	Failure	0.470	15.1	0.490	14.0	0.491	13.3
4	0.631	24.1	0.598	23.5	0.613	17.1	0.611	19.7
5	0.618	26.2	0.601	28.4	0.606	19.5	0.582	19.4
6	0.620	25.8	Failure	Failure	0.616	19.8	0.582	21.5
7	0.959	67.4	Failure	Failure	0.879	21.4	0.867	26.9
8	Failure	Failure	0.949	72.8	0.933	26.9	Failure	Failure
9	0.970	64.3	0.979	54.9	0.976	39.5	0.923	45.1
10	0.531	12.2	0.535	12.7	0.531	11.4	0.533	9.7
11	0.515	14.5	Failure	Failure	0.528	15.5	0.532	14.6
**12**	**0.487**	**7.6**	**0.471**	**11.1**	**0.496**	**9.9**	**0.483**	**12.3**
13	0.820	48.3	Failure	Failure	0.819	21.7	0.779	24.2
14	0.872	63.3	Failure	Failure	0.835	22.4	0.862	20.7
15	1.076	63.9	1.069	63.3	Failure	Failure	1.026	43.9
16	1.046	79.9	1.057	74.0	1.049	28.3	1.044	21.8
17	1.238	104.4	1.206	95.2	1.274	34.1	1.258	27.7
18	1.330	105.4	1.339	107.9	1.413	70.4	1.352	31.5

**Table 4:** Average stretchability and planar stretchability (PS) of the tested interconnects.

			Mechanical				Electrically			
	Theoretical		Free Standing		Encapsulated		Free Standing		Encapsulated	
Design	s [%]	PS [×1000]	s [%]	PS [×1000]	s [%]	PS [×1000]	s [%]	PS [×1000]	s [%]	PS [×1000]
1	8	13	37	62	39	64	33	55	28	47
2	5	18	39	134	36	123	32	108	22	75
3	5	24	36	163	37	169	32	147	29	133
4	32	27	58	49	55	46	51	43	39	33
5	32	51	68	108	52	83	58	92	41	66
6	32	72	65	146	49	110	55	123	44	99
7	107	47	170	76	67	30	143	64	51	23
8	107	90	150	127	81	69	155	131	57	49
9	107	131	128	157	92	113	127	156	90	110
10	5	35	27	178	31	202	26	172	22	146
11	11	20	51	93	44	82	31	57	32	59
**12**	**0**	**0**	**31**	**438**	**32**	**459**	**20**	**284**	**24**	**337**
13	67	26	112	44	57	22	103	40	49	19
14	84	43	131	67	69	35	135	69	46	23
15	122	193	137	216	99	157	135	214	93	148
16	123	50	185	76	64	26	164	67	53	22
17	165	108	223	146	91	60	212	139	66	43
18	184	164	228	203	104	93	227	203	108	97

**Table 5:** Compensated planar stretchability (CPS) ×1000.

		Mechanical		Electrical	
	Theoretical	Free Standing	Encapsulated	Free Standing	Encapsulated
Design	CPS [×1000]	CPS [×1000]	CPS [×1000]	CPS [×1000]	CPS [×1000]
1	4	21	21	18	16
2	3	22	21	18	13
3	3	18	19	16	15
4	9	16	15	14	11
5	24	18	14	15	11
6	16	16	12	14	11
7	16	25	10	21	8
8	15	21	11	22	8
9	15	17	13	17	12
10	2	11	13	11	9
11	5	23	20	14	15
**12**	**0**	**438**	**459**	**284**	**337**
13	13	22	11	20	10
14	14	22	12	23	8
15	15	17	12	16	11
16	17	25	9	22	7
17	18	24	10	23	7
18	18	23	10	23	11

## Supplementary Materials

The following are available online at http://www.mdpi.com/2072-666X/9/8/382/s1, Figure S1: AutoCAD circuit design file for the 60-m meander, Figure S2: AutoCAD circuit design file for the meanders tested using the tensile tester.
