# Supplementary material for: Stretchability—The Metric for Stretchable Electrical Interconnects
Source: Micromachines (Basel). 2018 Aug 1;9(8):382. doi: 10.3390/mi9080382 (PMC6187273; doi:10.3390/mi9080382)
Supplement: Supplementary file 1 [file micromachines-09-00382-s001.pdf]

micromachines

Supplementary Materials: Stretchability—The Metric for Stretchable Electrical Interconnects

Bart Plovie, Frederick Bossuyt and Jan Vanfleteren

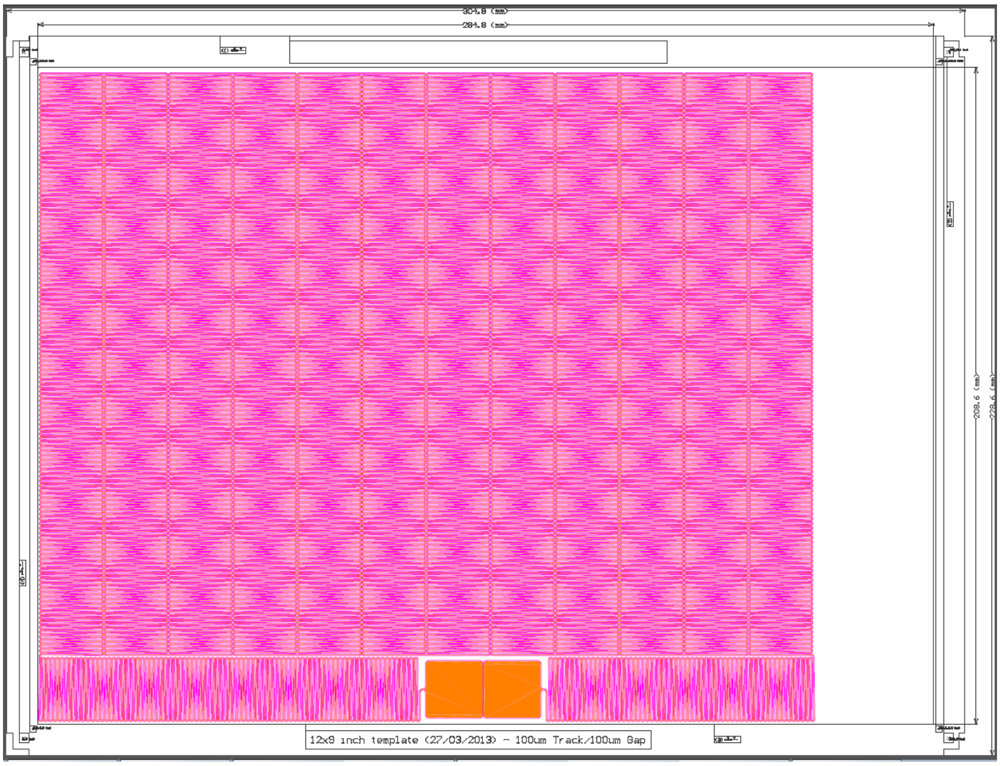

Figure S1. AutoCAD circuit design file for the 60-meter meander.

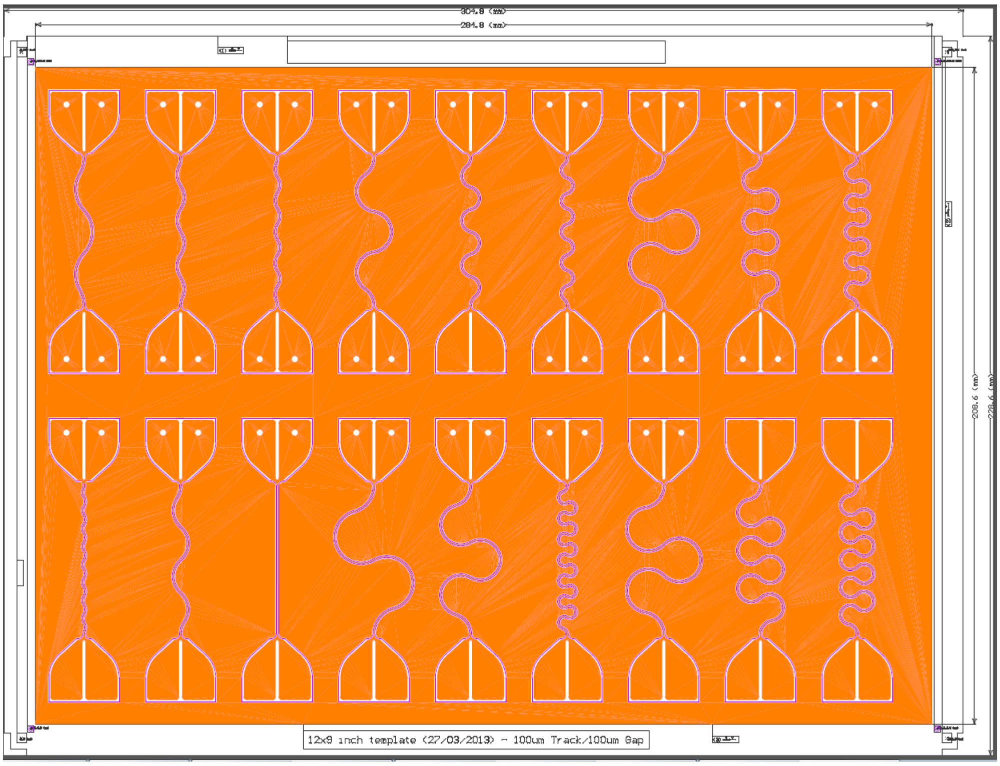

Figure S2. AutoCAD circuit design file for the meanders tested using the tensile tester.
